# Supplementary material for: PLA2R1 Inhibits Differentiated Thyroid Cancer Proliferation and Migration via the FN1-Mediated ITGB1/FAK Axis
Source: Cancers (Basel). 2023 May 11;15(10):2720. doi: 10.3390/cancers15102720 (PMC10216500; doi:10.3390/cancers15102720)
Supplement: Supplementary file 1 [file cancers-15-02720-s001.zip › Additional file S1.pdf]

Additional file S1:

Tables S1. Primer sequences for qRT–PCR.

Gene	Primer sequences
GAPDH	Forward: 5′-GGAGCGAGATCCCTCCAAAAT-3′
	Reverse: 5′- GGCTGTTGTCATACTTCTCATGG -3′
PLA2R1	Forward: 5′-TAAATCGGTTCTGACCCTGGA-3′
	Reverse: 5′-GCCACCGTAAGGAAACGAG-3′
ITGA1	Forward: 5′-CTGGACATAGTCATAGTGCTGGA-3′
	Reverse: 5′- ACCTGTGTCTGTTTAGGACCA -3′
ITGB1	Forward: 5′- CCTACTTCTGCACGATGTGATG -3′
	Reverse: 5′-CCTTTGCTACGGTTGGTTACATT-3′
ITGB4	Forward: 5′-GCTTCACACCTATTTCCCTGTC-3′
	Reverse: 5′-GACCCAGTCCTCGTCTTCTG -3′
ITGB6	Forward: 5′-GAGGACTACCCGGTGGATTTG-3′
	Reverse: 5′- TCCTTTATTGTGTTGAGGTCGTC-3′
FN1	Forward: 5′- AGGAAGCCGAGGTTTTAACTG -3′
	Reverse: 5′- AGGACGCTCATAAGTGTCACC -3′

Tables S2. Primary antibodies for Western blot analysis.

Antibody	Concentration	Company
PLA2R1	1:1000	Abcam
ITGB1	1:1000	Abcam
p-FAK	1:1000	Cell Signaling Technology
FAK	1:1000	Proteintech
E-cad	1:1000	Proteintech
N-cad	1:1000	Cell Signaling Technology
Cleaved-caspase3	1:1000	Cell Signaling Technology
caspase3	1:1000	Beyotime
GAPDH	1:20000	Cell Signaling Technology
Anti-mouse IgG (HRP)	1:5000	Cell Signaling Technology
Anti-rabbit IgG (HRP)	1:5000	Cell Signaling Technology

Anti-Snail

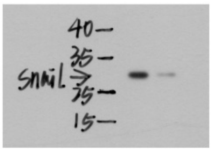

Anti-Slug

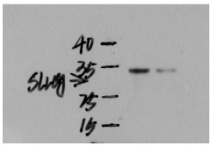

Anti-GAPDH

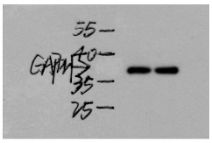

Anti-Snail

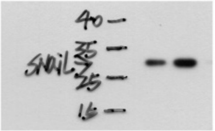

Anti-Slug

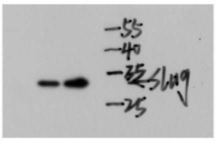

Anti-GAPDH

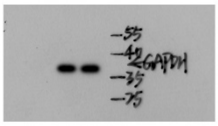

IB:ITGB1

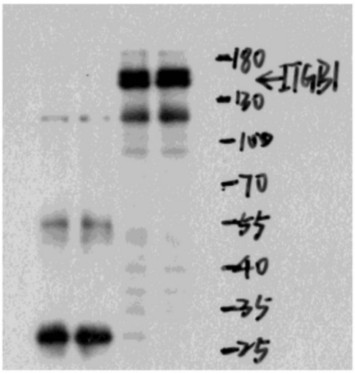

IB:FN1

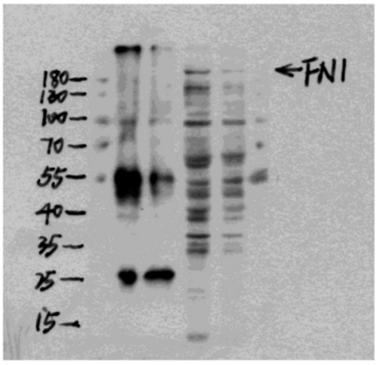

Anti-PLA2R

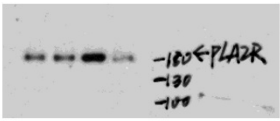

Anti-ITGB1

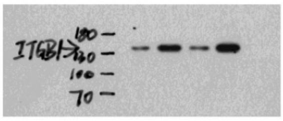

Anti-p-FAK

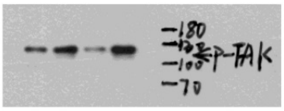

Anti-FAK

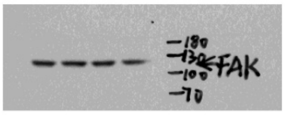

Anti-GAPDH

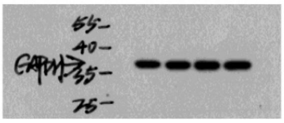

Anti-E-cadherin

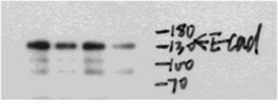

Anti-N-cadherin

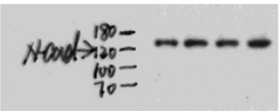

Anti-caspase3

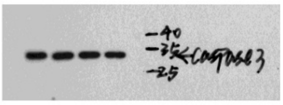

Anti-Clesved-
caspase3

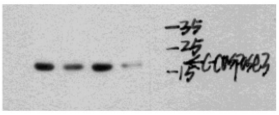

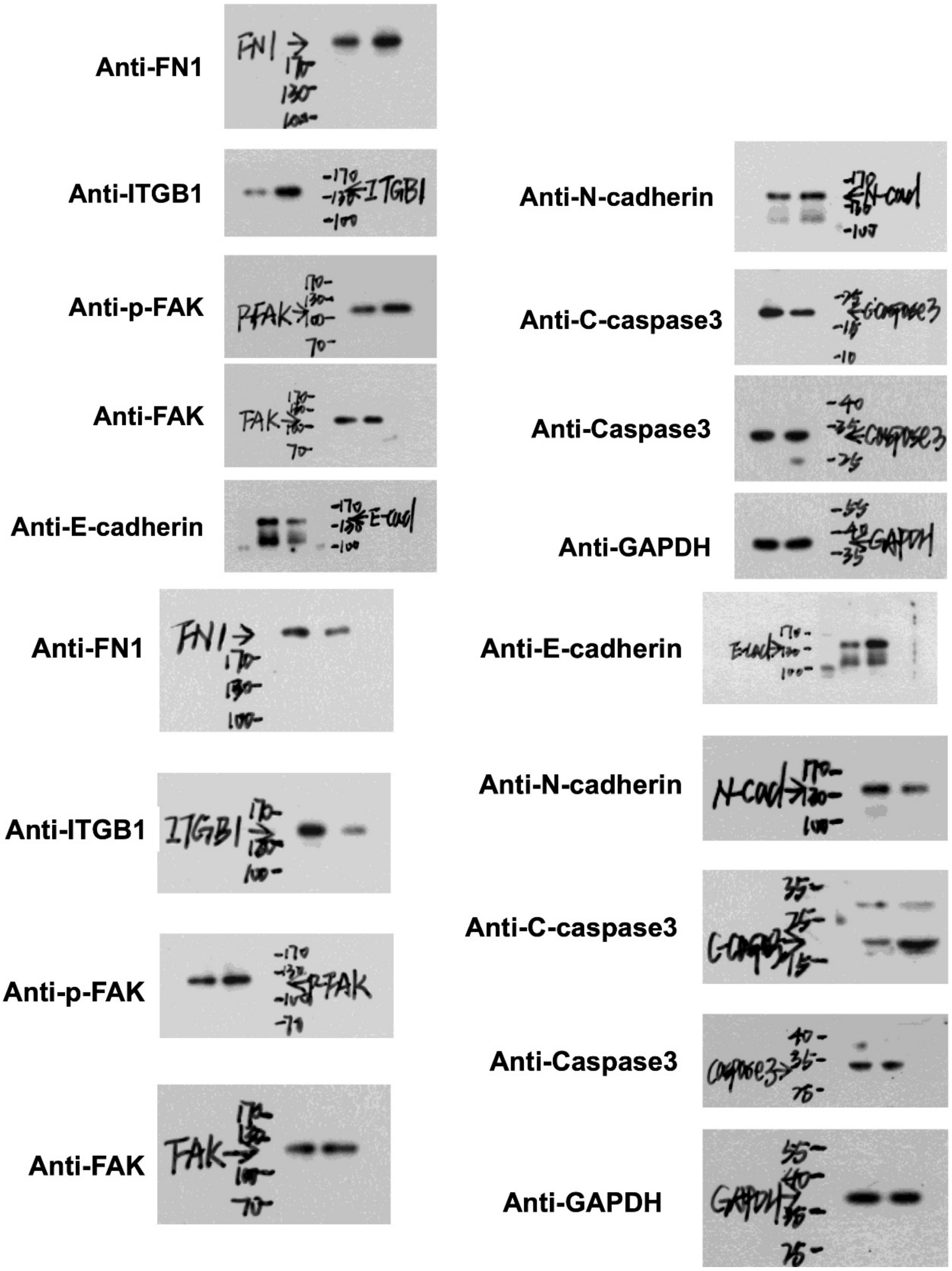
Anti-FN1
Anti-ITGB1
Anti-p-FAK
Anti-FAK
Anti-E-cadherin
Anti-N-cadherin
Anti-C-caspase3
Anti-Caspase3
Anti-GAPDH
Anti-FN1
Anti-ITGB1
Anti-p-FAK
Anti-FAK
Anti-E-cadherin
Anti-N-cadherin
Anti-C-caspase3
Anti-Caspase3
Anti-GAPDH
